# Alternative Prey and Artificial Diet of the Multicolored Asian Lady Beetle *Harmonia axyridis*: A Review

**DOI:** 10.3390/insects17010016

**Published:** 2025-12-23

**Authors:** Qingqiu Zeng, Rongrong Gao, Lamei Zheng, Kun Xue, Zhentao Ren

**Affiliations:** 1 Key Laboratory of Ecological Environment in Ethnic Areas, The State Ethnic Affairs Commission, Minzu University of China, Beijing 100081, China; 22011850@muc.edu.cn (Q.Z.); 22011979@muc.edu.cn (R.G.); 2 College of Life and Environmental Sciences, Minzu University of China, Beijing 100081, China; 3 Faculty of Life Science and Technology, Kunming University of Science and Technology, Jingming South Street, Kunming 650224, China; zhenglm100@163.com; 4 Institute of Food and Nutrition Development, Ministry of Agriculture and Rural Affairs, Beijing 100081, China

**Keywords:** *Harmonia axyridis*, artificial diets, rearing methods, alternative prey

## Abstract

As an important natural enemy insect, *Harmonia axyridis* has significant biological control effects on a variety of pests such as aphids in agroforestry ecosystems. The large-scale application of *H. axyridis* has long been limited by the unstable supply of natural prey, poor rearing of alternative prey and artificial diets, and high costs. In this paper, we review the research progress on the effects of alternative prey and artificial diets, and present the results and shortcomings of *H. axyridis* rearing. Focusing on the nutritional requirements of *H. axyridis* and the nutrient composition of natural prey, the paper aims to analyze and optimize the core components of artificial diets, develop diet formulations that are highly efficient, low-cost, support the ladybird throughout its complete life cycle, and identify the key factors for breaking the bottleneck of large-scale reproduction of *H. axyridis*, with a view to facilitate the industrialization of *H. axyridis*, enhancing the efficacy of biocontrol, and providing references to the development of diets for other natural enemy insects.

## Simple Summary

*Harmonia axyridis* is a vital natural predator capable of preying upon numerous agricultural pests and are in high demand for biological control. However, large-scale provision of these beetles currently relies heavily on rearing live aphids as a food source, a method characterized by high costs, low efficiency, and unstable supply, thereby limiting the beetles’ widespread adoption. To overcome this bottleneck, we comprehensively reviewed and analyzed extensive literature on artificial diets for lady beetles. Statistical evaluations were conducted on the rearing efficacy of diverse feed formulations. Our work aims to identify key determinants of effective artificial diets, providing a solid foundation for optimizing rearing protocols and enabling large-scale propagation.

## 1. Introduction

The multicolored Asian lady beetle, *Harmonia axyridis* (Coleoptera, Coccinellidae) is native to Asia. Its natural range extends westward to Kazakhstan, eastward to the Pacific coast, northward to southern Siberia, and southward to southern China [1]. *H. axyridis* primarily preys on aphids [2], but it also feeds on other major agricultural pests such as spider mites, scale insects, and whiteflies [3,4,5]. When prey is scarce, pollen and fruits can serve as alternative food sources [6]. As a predatory natural enemy, this species plays an important role in agricultural ecosystems [7,8]. With over a century of use in biological control, *H. axyridis* has been widely applied for pest management in orchards, greenhouses, croplands, and other agricultural environments [9,10,11,12]. In 1916, it was first released in North America as a biological control agent [13], where it achieved remarkable success in suppressing pests in pecan, apple, and citrus orchards [14,15,16]. After being introduced into Europe in 1964, it was tested for the biological control of various aphid species [17,18,19]. By 1995, *H. axyridis* had entered commercial use, with several biological control companies in France, Belgium, and the Netherlands beginning to market it [20]. Meanwhile, released populations of *H. axyridis* in Europe and North America have competed with native ladybirds [21,22]. Due to its superior overwintering ability, reproductive capacity, and predatory efficiency, it has been listed as an invasive species in Central Europe and North America [23,24], where it can outcompete native species preying on aphids [25,26]. Concurrently, *H. axyridis* preys upon *Adalia bipunctata* (Coleoptera, Coccinellidae) and *Adalia decempunctata* (Coleoptera, Coccinellidae) [27], as well as *Episyrphus balteatus* (Diptera, Syrphidae) [28]. Compared to native ladybirds, they are less frequently attacked by local parasitic wasps and pathogens [29,30]. Multiple factors have contributed to their rapid spread in invaded regions. In China, *H. axyridis* has been extensively employed to control pests in crops, vegetables, fruit trees, and forestry. It effectively suppresses aphid populations in cotton, tobacco, peach, apple, and protected vegetable fields [9,10,11,12,31], and provides excellent control of forest pests such as *Mindarus keteleerii* (Hemiptera, Aphididae), *Psylla chinensis*, and *Matsucoccus matsumurae* (Hemiptera, Margarodidae) [32,33,34].

Research on diets for *H. axyridis* has a history of more than 60 years, covering a wide range of topics including the exploration of alternative prey diets, optimization of artificial diet formulations, large-scale rearing techniques, and strategies for low-temperature storage. At present, studies on artificial diets and their application in ladybird rearing still face several limitations: (1) It is difficult for artificial diets to match natural prey in terms of nutritional balance and palatability, and cannot fully replicate the complex nutritional requirements provided by natural prey; (2) Most artificial diets cause a significant reduction in oviposition and require supplementation with natural prey to improve egg production; (3) Some diets induce elytral deformities or lead to a prolonged pre-oviposition period; (4) The cost of using alternative prey as diet remains high.

In this paper, we provide a review of the literature on artificial diets for the *H. axyridis*, with a particular focus on recent advancements. We summarize and consolidate successful diets and new methods for large-scale rearing of ladybirds using these diets. Based on a systematic review of recent research findings, we analyze the main technical bottlenecks in the development of artificial diets for *H. axyridis* and discuss potential pathways to overcome these limitations. The aim of this paper is to comprehensively and systematically review the formulation design, ingredient optimization, and rearing methods for artificial diets, while also exploring the existing challenges and possible strategies for improvement.

## 2. Artificial Diets for *Harmonia axyridis*

The survival rate and lifespan of *H. axyridis* reared on artificial diets have approached or even exceeded those fed natural prey, although their development duration and oviposition remain inferior to those fed natural prey.

### 2.1. Alternative Prey

**Figure 1:**
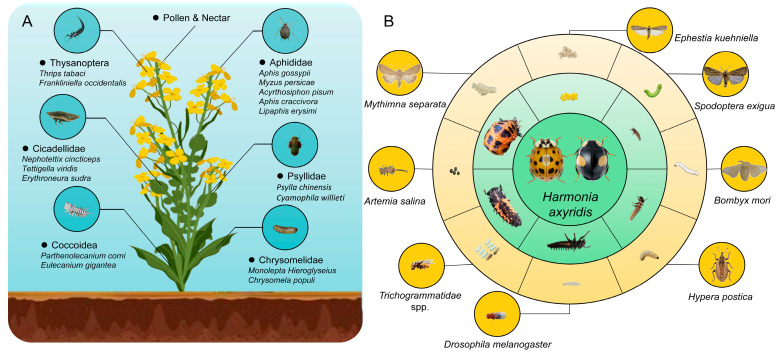
Summarizing multiple important crop pests preyed upon by *Harmonia axyridis*. Understanding the alternative prey options for *Harmonia axyridis*: (**A**) food sources of *Harmonia axyridis* in ecosystems, and (**B**) alternative prey selection in artificial rearing of *Harmonia axyridis*.

Natural prey of *H. axyridis* include *Aphis gossypii* (Hemiptera, Aphididae), *Thrips tabaci* (Thysanoptera, Thripidae), *Psylla chinensis* (Hemiptera, Psyllidae), and others (Figure 1A). Alternative prey species for captive rearing of *H. axyridis* primarily include: Lepidoptera such as *Ephestia kuehniella* (Lepidoptera, Pyralidae), *Corcyra cephalonica* (Lepidoptera, Pyralidae), *Sitotroga cerealella* (Lepidoptera, Gelechiidae), and *Pectinophora gassypiella* (Lepidoptera, Gelechiidae); Hymenoptera such as *Trichogrammatidae* spp. (Hymenoptera, Trichogrammatidae); Coleoptera such as *Hypera postica* (Coleoptera, Curculionidae); and Anostraca such as *Artemia salina* (Anostraca, Artemiidae) (Table 1). 

We summarize and provide examples of alternative prey species reported in the literature (Figure 1B). In the research on alternative prey diets, Mediterranean flour moth eggs have been the most studied and widely used, followed by effective results with Oriental tobacco budworm and beet armyworm, all of which can be used for large-scale rearing of *H. axyridis* (Figure 1).

#### 2.1.1. *Ephestia kuehniella*

*Ephestia kuehniella* has been extensively studied as an alternative prey capable of meeting the nutritional requirements of various predatory ladybird, such as *Adalia bipunctata* (Coleoptera, Coccinellidae), *Propylea japonica* (Coleoptera, Coccinellidae), and *Semiadalia undecimnotata* (Coleoptera, Coccinellidae) [35,49,50]. As early as 1972, research demonstrated that eggs of *E. kuehniella* could support the complete development of the aphidophagous ladybird *Ceratomegilla undecimnotata* [51]. In the same year, another study confirmed that *E. kuehniella* eggs could be used to rear *H. axyridis*, although individuals fed with these eggs showed a longer developmental period and lower body weight compared with those fed aphids [52]. Starting in 1986, *H. axyridis* was successfully reared for more than 100 generations on ultraviolet-irradiated *E. kuehniella* eggs [53]. In 1988, it was found that UV-treated eggs preserved at low temperatures significantly increased the oviposition rate of *H. axyridis*, though the hatch rate of its eggs declined [35]. Further studies revealed that compared with *Aphis gossypii* and the eggs of *Sitotroga cerealella*, *E. kuehniella* eggs resulted in shorter larval development times, heavier adult body weights, and higher oviposition and hatch rates in *H. axyridis* [37]. Similarly, when compared with cotton aphids or mixed diets, *E. kuehniella* eggs produced higher survival rates, shorter development times, and greater adult body weight and length [39]. However, relative to *Cinara atlantica* (Hemiptera, Aphididae) and *Brevicoryne brassicae* (Hemiptera, Aphididae), *E. kuehniella* eggs led to lower reproductive capacity and body weight [38]. Overall, most studies support the use of *E. kuehniella* eggs as a substitute for natural prey in rearing *H. axyridis*, though certain drawbacks have been noted; some research even suggests that these eggs are more suitable than *Acyrthosiphon pisum* (Hemiptera, Aphididae) or cotton aphids as food for *H. axyridis* [54].

*E. Kuehniella* eggs are commonly used as alternative prey for predatory insects, and numerous studies have confirmed their effectiveness, but the rearing outcome depends heavily on the supply and quality of the eggs [55]. To improve preservation, the eggs require special treatment; freezing for one day typically yields the best rearing results for ladybirds [56]. However, such procedures are labor-intensive and may affect storage stability, and the rearing performance still does not match that obtained with live aphids. Ferran et al. found that larvae of *H. axyridis* reared on *E. kuehniella* eggs exhibited weaker prey-tracking ability when subsequently offered aphids than larvae reared directly on aphids [57]. Likewise, *H. axyridis* larvae maintained for 100 generations on *E. kuehniella* eggs showed difficulty locating aphids and had lower predation efficiency [53]. In contrast, Sun et al. observed that adults of *H. axyridis* reared on *E. kuehniella* eggs did not display a significant reduction in searching or attacking behavior toward *Aphis craccivora* (Hemiptera, Aphididae), especially when the diet was supplemented with honey, which produced performance most similar to that of aphid-fed individuals [58]. In summary, properly treated *E. kuehniella* eggs provide stable nutritional value and can serve as a substitute for natural prey in rearing *H. axyridis*. However, the cost remains relatively high, and long-term rearing on *E. kuehniella* eggs carries a risk of functional decline in predatory ability.

#### 2.1.2. Other Lepidoptera Insects

Following studies on Mediterranean flour moth eggs, researchers have found that *Spodoptera exigua* (Lepidoptera, Noctuidae) and *Mythimna separata* (Lepidoptera, Noctuidae) also serve as effective diets for rearing *H. axyridis*. These two alternative prey species have already been applied in large-scale *H. axyridis* cultivation. Wang et al. evaluated the rearing performance of four mass-reared lepidopteran larvae—*S. exigua*, *Spodoptera litura* (Lepidoptera, Noctuidae), *Helicoverpa armigera* (Lepidoptera, Noctuidae), and *Plutella xylostella* (Lepidoptera, Plutellidae)—and found that early instar larvae of *S. exigua* successfully supported large-scale production of *H. axyridis* [59]. Compared with the other three lepidopteran species, early instar *S. exigua* larvae exhibited several advantages: they feed on the diet surface, display moderate mobility, distribute evenly in ladybird rearing containers, lack aggressiveness, and are highly palatable [59]. Huang et al. reared *Coccinella transversalis* (Coleoptera, Coccinellidae) on early instar *S. exigua* larvae and showed that the diet fully supported ladybird growth and development; however, the pre-oviposition period was prolonged and egg production decreased [60]. Wu et al. demonstrated that *M. separata* eggs are an excellent diet for *H. axyridis*, supporting its complete life history with high survival rates. Furthermore, supplementing with sugar sources boosted reproduction, which facilitated the successful mass production of the beetle continuously over multiple generations at high densities [46,61]. Currently, *S. exigua* and *M. separata* represent superior alternative prey for the large-scale propagation of *H. axyridis* and hold significant application potential. However, it remains unclear whether long-term rearing of *H. axyridis* on *S. exigua* might lead to population degeneration. Additionally, the production cost of using *M. separata* eggs remains relatively high.

In contrast, other Lepidoptera insects exhibit poorer rearing results. Study has shown that Angoumois grain moth eggs can be used as a supplemental food source for *H. axyridis* [62] The earliest attempt involved supplementing overwintering ladybirds with a mixture of chicken egg, tussah pupa slurry, sugar, and water in specific proportions, which increased post-winter survival and prolonged adult lifespan [63]. While both fresh and frozen eggs are suitable, fresh eggs demonstrate superior performance in development time, survival rate, body weight, and fecundity [41]. Although pink bollworm eggs can support the growth, development, and reproduction of *H. axyridis*, the resulting adults are lighter and lay fewer eggs than those reared on grain moth eggs [44]. When silkworm larvae were used as food, larval developmental duration and adult body weight were similar to those of aphid-fed controls, but the pre-oviposition period was prolonged and egg production reduced [45].

#### 2.1.3. Other Alternative Prey

Researchers have also screened a variety of commonly mass-reared alternative prey, including the western honeybee, *Apis mellifera* (Hymenoptera, Apidae) and *Trichogrammatidae* spp.; the coleopteran alfalfa weevil, *Hypera postica*; the dipteran fruit fly, *Drosophila melanogaster* (Diptera, Drosophilidae); and the anostracan brine shrimp, *Artemia salina*. However, most of these prey showed unsatisfactory rearing performance for *H. axyridis* (Table 1).

When adult *H. axyridis* and *Propylea japonica* were fed solely on drone pupae powder, both species entered reproductive diapause [64]. Feeding *H. axyridis* with rice moth eggs or *Trichogramma* pupae revealed that rice moth eggs could not support complete metamorphosis, while *Trichogramma* pupae did allow for full development but resulted in a markedly prolonged developmental period and significantly reduced oviposition [40]. Artificial diets prepared from freeze-dried western honeybee larvae could sustain ladybird development, but their effectiveness was clearly inferior to that of natural aphid prey [62]. Feeding *H. axyridis* larvae with artificial eggs plus *Trichogramma* pupae, tussah eggs plus *Trichogramma*, or drone pupae showed that all three diets could serve as larval substitutes. Among them, the combination of artificial eggs and *Trichogramma* pupae performed best: adult emergence rate and larval development time were not significantly different from those of aphid-fed controls, yet the pre-oviposition period, oviposition rate, total egg production, and egg hatchability were all significantly lower [65]. Rearing *H. axyridis* with *Chouioia cunea* (Hymenoptera, Eulophidae) pupae enables them to complete their entire life cycle within a relatively short period, and the egg hatching rate reaches as high as 97.37%, while egg production and oviposition cycles are slightly lower than when reared with aphids [66]. First-instar *H. axyridis* larvae fed alfalfa weevil larvae failed to reach the second instar; fourth-instar larvae were able to pupate, but adult body weight was significantly lower than in aphid-fed controls [67]. Furthermore, adults reared on alfalfa weevils could not oviposit and weighed less than those reared on pea aphids [47]. Brine shrimp eggs supported complete development and oviposition; however, they also caused longer developmental times, significantly reduced pupal weight and a high incidence of wing deformities [48]. Fruit fly larvae supported full development, but adults exhibited mating behavior only and failed to lay eggs [68]. At present, all of these alternative prey species can serve only as nutritional supplements when natural food is scarce; they cannot adequately support growth and development nor enable large-scale reproduction of *H. axyridis*. From a cost perspective, brine shrimp eggs cost only about one-tenth as much as Mediterranean flour moth eggs, suggesting that if their shortcomings can be overcome, they hold considerable potential for large-scale application.

### 2.2. Artificial Diets

As early as in 1958 artificial diets containing natural prey powders were successfully used to rear multiple predatory ladybird species [69]. In 1977, a substitute diet containing honey and fresh pig liver homogenate was able to successfully rear *Coccinella septempunctata* (Coleoptera, Coccinellidae) and *H. axyridis* [70]. Over time, researchers developed more complex diets by incorporating plant proteins, carbohydrates, and lipids. Through continuous optimization, artificial diets primarily based on pig liver, eggs, and drone bee larvae can support complete development of ladybirds, although egg hatchability remains significantly lower than in aphid-fed groups [71]. Overall, the performance of *H. axyridis* reared on artificial diets has improved to varying degrees in terms of survival rate and developmental duration. However, fecundity and egg hatchability still lag substantially behind those achieved with natural prey (Table 2).

#### 2.2.1. Artificial Diets Based on Alternative Prey

When alternative prey cannot fully meet the nutritional requirements of *H. axyridis*, the addition of nutrients such as sugars can significantly improve rearing performance. Studies have shown that supplementing *Ephestia kuehniella* eggs with bee-collected pollen shortens the pre-oviposition period of *H. axyridis*, although other developmental and reproductive parameters remain unaffected [84]. Similarly, adding perilla (*Perilla frutescens*) pollen and nectar to *E. kuehniella* eggs had a positive, though non-significant, effect on survival and early reproduction [85]. Supplementing *Mythimna separata* eggs with glucose resulted in *H. axyridis* with a shorter developmental duration and significantly higher individual female fecundity compared to unsupplemented groups [86]. The addition of sugar sources triggered the first oviposition in *H. axyridis* fed on either young or older *M. separata* larvae, with honey being particularly effective [86]. A paste-like diet composed of drone bee pupae and honey supported *H. axyridis* development and reproduction comparable to aphid-fed controls [87]. Furthermore, *H. axyridis* adults reared on drone bee pupae received a daily application of 100 μg of juvenile hormone analog ZR-512 (Ethyl 3,7,11-trimethyldodeca-2,4-dienoate) for two days post-emergence. This treatment significantly increased their vitellin (Vn) content, which reached peak levels and rates similar to those of the aphid-fed controls. This treatment significantly increased food intake and egg production [64]. Using a diet of drone bee larvae and honey, individual females produced an average of 520.4 eggs [34]. *Hypera postica* larvae are generally poor prey for *H. axyridis* and do not support oviposition; however, mixing them with sugar water (15% sucrose solution) enabled the production of a small number of eggs [47]. Adding sucrose to *Artemia salina* eggs improved the previously low hatchability and high wing deformity rates observed when *H. axyridis* were reared solely on Artemia [60]. Supplementing the diet of *Drosophila melanogaster* larvae with sucrose, fructose, or honey improved larval survival and adult body weight and induced oviposition behavior [68]. In a nutshell, these studies indicate that adding sugars and other nutrients can partially compensate for the shortcomings of alternative-prey-based diets, but their effectiveness still falls short of that achieved with natural prey.

#### 2.2.2. Liver-Based Artificial Diets

Early studies successfully reared *H. axyridis* using artificial diets that were based on liver and supplemented with sucrose, honey, and other additives. However, these diets led to adverse outcomes including high mortality and prolonged development; they also resulted in adults with lower body weight, reduced fecundity, and poor egg hatchability [70,71]. Through optimization of ingredient ratios and the addition of functional substances, the performance of liver-based diets has been significantly improved. In 1982, it was reported that supplementing a pig-liver-based diet with silkworm pupal powder increased the fecundity of *H. axyridis* [72]. Artificial diets containing fly larval powder allowed larval survival rates to reach 96.67% and shortened the pupal period, though developmental duration remained long and adult body weight and reproductive capacity decreased [76]. Based on the diet developed by Yang et al., further optimization with the addition of glucose or trehalose shortened the pre-oviposition period and increased fecundity (by approximately 30%) and hatchability (approaching levels seen with natural prey) [77,88]. Optimized non-insect diets supported larval survival rates of up to 82.2%, but adult fecundity was only 5% of the aphid-fed group; however, if larvae were fed artificial diet and adults were switched to aphids, fecundity could recover to 80% of the control group [80]. Besides pig liver, chicken, and beef liver can also serve as the main ingredients in artificial diets. Dong et al. found that diets based on chicken liver, starch, and sucrose supported *H. axyridis* survival and adult body weight comparable to those fed *Sitotroga cerealella* eggs, though fecundity and developmental duration were lower. Moreover, diets containing whole eggs demonstrated better performance than those with only egg yolk in all measured parameters: survival, developmental duration, and body weight [44]. Ali et al. reared *H. axyridis* on a diet of beef liver, beef, shrimp, and egg yolk. They found that while developmental duration and adult body weight were slightly lower than in the *Acyrthosiphon pisum* group, both survival and fecundity were comparable to those reared on natural prey [83]. Currently, the performance of liver-based diets still lags behind aphid feeding. Prolonged consumption of artificial diets can suppress female reproductive development, including impaired ovary maturation and reduced vitellogenin (Vg) expression, resulting in longer pre-oviposition periods and lower fecundity. Studies have shown that reproductive performance reduced by artificial diets can be restored when adults are supplemented with aphids, with fecundity comparable to that of continuously aphid-fed adults [83,89]. Additionally, liver-based diets are rich in unsaturated fatty acids and prone to oxidation during long-term storage. Fresh pig liver also spoils easily at room temperature, and thus requires cold storage or preservatives such as potassium sorbate. Alternatively, pig liver powder can be used to replace fresh liver as an optimization strategy.

## 3. Nutritional Components and Improvement of *Harmonia axyridis* Artificial Diets

The composition of artificial diets must meet the basic nutritional requirements for insect growth, development, and reproduction, including carbohydrates, proteins, lipids, vitamins, and inorganic salts [90,91]. Studies have shown that the use of synergistic substances can improve the deficiencies of artificial diets [78,92]. The key breakthrough for *H. axyridis* artificial diets may lie in mimicking the complex nutritional profile provided by aphids, thereby compensating for the reduced reproductive capacity observed in current artificial rearing due to nutritional deficiencies. The primary nutritional component of aphids is crude protein. Additionally, they are rich in 10 essential amino acids, and their lipids consist predominantly of unsaturated fatty acids [93]. The honeydew of aphids consists primarily of sugars, which account for over 80% of its dry weight and include sucrose, glucose, and fructose [94]. Additionally, limonene has been identified as the most effective attractant for *H. axyridis* [95]. Investigating the effects of different nutritional components in artificial diets on *H. axyridis* provides important reference data for the development and improvement of such diets [96].

### 3.1. Basic Components

#### 3.1.1. Carbohydrates

Carbohydrates are one of the fundamental nutrients for insect growth and development and constitute an important component of artificial diets for insects [97]. Current research indicates that carbohydrates can sustain the basic survival of *H. axyridis* and, when added to a basal diet, can also reduce wing deformities and enhance reproductive performance. Certain sugars can act as precursors for polyols, serving as cryoprotectants during winter [98,99,100]. Galvan found that feeding *H. axyridis* with sucrose solution in autumn significantly improved overwintering survival compared to water-only or starved treatments [91]. Seko et al. reported that incorporating sucrose into the diet reduced the proportion of wing deformities, increased female body size, and improved egg hatchability [48]. Lundgren summarized that adult beetles can survive on sugar water alone, and adding sugar to diets containing natural prey can improve adult physiological traits and enhance reproduction [101]. Niijima et al. found that a fully artificial diet could support larval development from first to third instar, but failed to induce oviposition; however, supplementing the diet with water-soluble extracts from male bee pupae enabled egg-laying, suggesting that some carbohydrates and possibly other unknown compounds may play key roles [102].

Trehalose plays an important role in the development and stress response of *H. axyridis*. Jing et al. found that trehalose provides energy during starvation through molecular and biochemical regulation [103]. Zhang et al. demonstrated that injecting trehalase genes TER2dsTRE2-like and dsTRE2 into *H. axyridis* pupae disrupted trehalose metabolism, affecting glycogen and glucose supply, which may lead to wing deformities [104]. Wang et al. reported that trehalose metabolism plays a critical regulatory role under cold stress or cold storage, based on comparative analyses of gene expression and enzyme activity [95]. Li et al. showed that glucose and trehalose supplementation can enhance reproduction and support growth and development, whereas loss of glucose transporter 4 impairs ovary development and oocyte maturation, resulting in reduced reproductive capacity [77,105].

#### 3.1.2. Proteins and Amino Acids

Proteins and amino acids, as the primary structural components of insect bodies, constitute one of the essential material bases for insect growth and development [97]. A deficiency of amino acids or proteins in artificial diets can hinder insect growth and development [106]. Insects require both essential and non-essential amino acids. The essential amino acids generally include arginine, lysine, leucine, isoleucine, tryptophan, histidine, phenylalanine, methionine, threonine, and valine, although interspecies variations exist [107]. Vg is the precursor of Vn, the main storage protein in the eggs of many oviparous animals, and plays a crucial role in reproduction [108,109]. It is closely associated with the reproductive capacity of *H. axyridis* [110,111]. Zhang et al. reported that adding purified soluble Vg fragments to artificial diets of *H. axyridis* altered enzyme activities compared to the control group, thereby improving nutrient digestion from the artificial diet and enhancing reproductive performance [112,113]. Other studies in different insects have shown that Vg not only regulates reproduction but may also be involved in food storage, immune responses, and stress tolerance [114,115,116]. However, these additional functions still require further investigation in *H. axyridis* [114,115,116].

#### 3.1.3. Lipids and Sterols

Insect growth, development, and reproduction fundamentally depend on lipids and sterols, whose essential role in artificial diet development remains commonly underestimated [117]. The energy required for insect activity is stored in the form of glycogen and fats, making fatty acids and sterols important nutritional components in artificial diets for insects [117]. Fatty acids are the main constituents of insect body lipids. Insects require two types of fatty acids. The first type is saturated fatty acids, which *H. axyridis* can synthesize internally. For example, when reared on pea aphids, *H. axyridis* must convert some short-chain fatty acids (including myristic acid) into 18-carbon fatty acids [36]. The second type is unsaturated fatty acids, which are essential nutrients for many insects but must be obtained from food. Sighinolfi et al. further investigated the development of artificial diets with varying fatty acid content and found that total fatty acid content in *H. axyridis* remained unchanged. Moreover, even when flaxseed oil was added to the diet, the biological parameters of *H. axyridis* were still inferior to those reared on *E. kuehniella* eggs [73,75]. This likely does not simply indicate that fatty acids are “unimportant”, but rather suggests that the added lipid form was not efficiently utilized within the diet matrix or failed to meet *H. axyridis*’s specific requirements for certain lipid complexes [117]. This may suggest that diet success hinges not only on the presence of nutrients but critically on its organizational matrix—which directly determines nutrient accessibility and bioavailability [117]. Thus, the total fatty acid content alone is insufficient; the structure and presentation within the diet matrix are paramount for nutritional success.

Cholesterol, also known as a sterol, cannot be synthesized by insects and must be obtained from food to maintain normal growth and development. Measurements of sterol content across different developmental stages of *H. axyridis* indicate that newly hatched larvae may acquire relatively high concentrations of estradiol from their diet [118]. Additionally, *H. axyridis* is unique in its ability to readily assimilate ergosterol, obtaining sterols through the consumption of plant tissues, which improves their overall health [119].

#### 3.1.4. Inorganic Salts

Approximately half of all successful insect diets incorporate salt mixtures, yet research into their specific roles in insect nutrition remains limited [117]. Among these, potassium salts have been identified as an essential inorganic component in artificial diets for *H. axyridis*. Matsuka et al. analyzed the composition of dried male bee powder and highlighted the functional role of inorganic salts, confirming that *H. axyridis* larvae have a specific requirement for minerals—especially potassium—during growth and development [120]. Exposure to excessive NaCl can hinder the accumulation of metabolites within *H. axyridis*, particularly several sugars, amino acids, organic acids, and fatty acids [121]. 

### 3.2. Nutrients for Improving Rearing Quality

**Figure 2:**
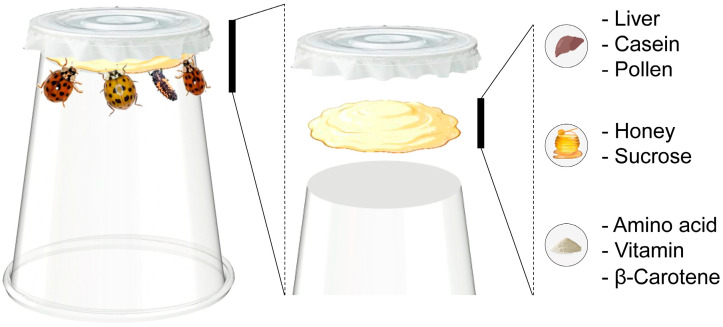
An artificial diet device for raising *Harmonia axyridis* and nutrient-specific improvements.

The use of artificial diet containing high-quality nutrients and scientifically designed rearing apparatus can better ensure the growth and development of *H. axyridis*. An effective device for rearing *H. axyridis* involves placing artificial diet at the base of disposable plastic cups, covering the center with a small piece of cling film or wax paper, and leaving the periphery accessible for feeding (Figure 2) [122]. This setup offers three principal advantages: (1) Preventing the diet from drying out and clumping, (2) avoiding ladybirds becoming trapped and perishing, (3) and concentrating the feeding area to improve utilization. Fresh diet must be replaced daily. Once the peripheral diet has been largely consumed, the central covering may be removed. Currently, effective synergistic substances that have been confirmed include carbohydrates such as glucose, pollen and honey sources, Vg, juvenile hormone, and *β*-carotene [86,123].

#### 3.2.1. Pollen and Nectar

Pollen is rich in sugars, proteins, lipids, amino acids, sterols, and various vitamins, making it nutritionally suitable for many life processes of beneficial insects [124]. In corn fields, a small number of *H. axyridis* larvae were found to contain corn pollen in their guts, indicating that *H. axyridis* will consume small amounts of pollen even when natural prey is available [125].

Adding *Cnidium monnieri* flowers to a diet based on aphids can improve the developmental time, survival, and adult body weight of *H. axyridis* larvae, suggesting that the pollen and nectar in the flowers contribute to their growth [126]. Compared with the control group fed only aphids, feeding *Coriandrum sativum* flowers increased reproductive capacity, whereas feeding *Calendula officinalis* reduced reproduction by 22% [127]. When *Perilla frutescens* flowers were added, *H. axyridis* showed significantly higher first-day egg production compared with individuals fed only *Ephestia kuehniella* eggs, though total long-term egg production and oviposition frequency were not significantly affected. This suggests that *P. frutescens* may accelerate ovarian maturation by providing additional nutrition in the presence of natural prey but cannot replace prey as a necessary food source [85]. Compared with feeding only *Myzus persicae*, the addition of *Brassica napus* pollen and nectar significantly increased adult egg production and extended lifespan, while having no significant effect on larval predation, survival rate, or developmental time. *B. napus* pollen was also shown to be more attractive to *H. axyridis* [123]. Similarly, supplementation with peach blossom nectar increased survival rates by 60% compared with aphid-only diets [128].

When providing plant-based food sources, feeding corn pollen alone allowed approximately 50% of larvae to develop into adults and reproduce [129]. Larvae fed artificial diets supplemented with pollen had significantly higher survival to adulthood than control diet groups, although developmental time and adult body mass were not significantly improved. Pollen species and concentration had a clear effect on larval growth, with high-concentration rose pollen and medium-concentration corn pollen producing the best results [130]. Further studies showed that adding *B. napus* pollen to artificial diets significantly improved larval development and adult quality, and supported pupal-to-adult metamorphosis by regulating nutrient metabolism, immune responses, and gene expression related to epidermal development [78]. *B. napus* pollen with 23.1% water content was most favorable for larval growth and significantly improved survival and reproduction under conditions of insufficient *M. persicae* [131]. Berkvens et al. found that adding frozen bee-collected pollen to a diet of *E. kuehniella* eggs advanced the oviposition period, and diets consisting solely of frozen bee pollen could also support development to adulthood [84].

In summary, when prey is abundant and of high quality, supplementing pollen has limited effect on *H. axyridis*, but adding nectar can enhance rearing outcomes. When prey is scarce, of low quality, or absent, pollen supplementation is more effective. Pollen species, concentration, and water content are critical factors. It is important to note that nutritional value varies among species and within species, thus actual effects cannot be generalized [132].

#### 3.2.2. Casein and Milk Powder

Casein is commonly added as a mass nutritional component and an exogenous substitute for animal protein in most successful insect artificial diets [117]. Its inclusion is supported by a complete amino acid profile and high digestibility, which have also shown significant potential in ladybird artificial diets. Studies have found that providing casein in *Coccinella septempunctata* artificial diets can support oviposition and achieve good rearing outcomes [133,134]. Compared with casein, adding casein hydrolysates to the diet further improves larval rearing, indicating that larvae can more fully absorb and utilize amino acids or peptides. This effect may also be related to the lack of certain corresponding digestive enzymes in the larvae [135].

Milk powder and skimmed milk powder are included in many optimized formulations. They provide proteins, carbohydrates, and fat-soluble vitamins, serving both nutritional and carrier functions in ladybird diets. Their effectiveness has been demonstrated in the rearing of *C. septempunctata* and *H. axyridis* [83,134]. In formulations with a higher proportion of milk powder, all biological parameters of *C. septempunctata* showed good performance [136]. Further research found that adding milk powder containing small amounts of lipids, as well as other lipid substances, can improve larval survival rates [137].

#### 3.2.3. Juvenile Hormone

Juvenile hormone (JH) is an important regulator of female reproduction [138]. Studies have shown that ladybirds fed on artificial diets exhibit slow ovarian development, but adding different concentrations of JH to the artificial diet can improve ovarian development to varying degrees, alleviating the low reproductive capacity observed in populations reared on artificial diets [139]. Further research has found that the application of JH analogs can slightly shorten the pre-oviposition period and increase oviposition [70]. Gao et al. reported that reducing JH titers induces reproductive diapause in *H. axyridis*, and JH analogs could serve as potential insect growth regulators to control diapause in this species [140].

#### 3.2.4. Carotenoids

*β*-Carotene serves as a precursor for certain vitamins and hormones and functions as an antioxidant in organisms [141]. *H. axyridis* cannot synthesize *β*-carotene endogenously and must acquire it from its diet, whereas aphids possess genes enabling *β*-carotene biosynthesis [142]. Studies have shown that supplementing artificial diets with *β*-carotene can bring larval survival rate, egg hatchability, and pre-oviposition period of *H. axyridis* close to those of individuals fed natural prey, although developmental duration and fecundity remain lower than in aphid-fed groups [143]. Lu et al. reported that *C. septempunctata* larvae reared on an artificial diet containing 50 mg/kg *β*-carotene exhibited significantly higher pupation and adult emergence rates compared to other diet combinations, approaching the performance of aphid-fed controls [144]. Lin et al. tested *β*-carotene supplementation at 15, 20, and 25 mg/kg, and found that increased *β*-carotene levels darkened egg coloration and maximized oviposition at 20 mg/kg [145]. These results indicate that *β*-carotene supplementation can enhance ladybird rearing performance, potentially through its role in vitamin A synthesis, although the precise mechanisms remain unclear.

#### 3.2.5. Nutritional Composition of Aphids and Aphid Honeydew

Currently, artificial diets for H. axyridis still show deficiencies in nutritional composition and rearing outcomes, particularly regarding reproductive performance, compared to natural prey. Therefore, systematic optimization based on the nutritional characteristics of aphids and their honeydew is crucial. Aphids, as the primary prey of H. axyridis, are rich in sugars, high-quality proteins, lipids, essential amino acids, and vitamins [93]. Aphid honeydew contains high concentrations of carbohydrates along with free amino acids, trace vitamins, minerals, and secondary metabolites [146], with some components varying depending on the host plant [147] (Table 3 and Table 4). By mimicking specific components of aphid honeydew, artificial diet formulations can be further improved. Optimized diets demonstrate superior nutritional balance, palatability, and promotion of ladybird growth and development compared to conventional formulations, bringing their performance closer to that achieved by natural prey.

## 4. Summary and Discussion

This review systematically summarized the research progress on *H. axyridis* artificial diets, focusing on three aspects: the development of alternative prey-based diets, the rearing performance of artificial diets, and the optimization of nutritional components. The development of artificial diets reduces reliance on natural prey, provides a stable insect source for biological control, enables large-scale production, accelerates the commercialization of natural enemies, promotes green agriculture, and reduces the cost of field biological control. Existing studies indicate that artificial diets have the potential to partially replace natural prey in large-scale rearing. However, their nutritional deficiencies, poor palatability, and reproductive suppression still limit practical applications. Although diet formulations improved according to insect nutritional requirements and supplemented with nutrients that enhance rearing quality can significantly improve larval survival and adult longevity, technical challenges remain. These include the absence of key reproductive regulatory factors in artificial diets, mismatches between the nutritional composition of artificial diets and the nutritional needs of *H. axyridis*, the potential reduction in predatory capacity following several generations of mass rearing on artificial diets, and the as-yet-unclear synergistic mechanisms of feeding stimulants [76]. It should also be stressed that successful diet development depends on a functional diet matrix—one that ensures both accessibility (i.e., adaptation to insect feeding morphology and sensory cues, presented in an ingestible form) and bioavailability (i.e., nutrient forms that facilitate digestion and absorption)—beyond merely providing required nutrients [117].

The development and reproduction of *H. axyridis* are highly dependent on complex nutrient supply. Key dietary components for successful rearing include trehalose, pollen and nectar, amino acids, fatty acids, sterols, *β*-carotene, feeding stimulants such as limonene, and developmental regulators such as juvenile hormone analogs and vitellogenin. These studies provide crucial data on nutrient metabolism, improved rearing quality, artificial diet formulation optimization, and large-scale rearing, offering theoretical and technical support for the industrialization of natural enemies. In particular, during seasonal shortages of natural prey, improvements in artificial diets can significantly enhance the stability and economic efficiency of biological control with *H. axyridis*.

It is worth noting that *H. axyridis*, due to its eurytopic nature, serves as an excellent biological control agent whilst also posing certain ecological risks when released into the wild. In Europe, North America, and other introduced regions, *H. axyridis* possesses competitive advantages in life history parameters such as development cycle and reproductive capacity [21,22]. As a potent intraguild predation (IGP) predator, it may displace native ladybird species (such as the two-spotted ladybird) from their ecological niches, thereby altering community structure [149]. Their broad-spectrum feeding behavior extends beyond pests—studies indicate they prey on other arthropods when primary prey is scarce. Koch et al. highlighted potential negative impacts on monarch butterflies, *Danaus plexippus* (Lepidoptera, Nymphalidae) [150]. Even when primary prey is present, a certain quantity of other insects will also be consumed [151]. Regarding impacts on human activities, *H. axyridis* congregate in North American vineyards, and their reflex bleeding during grape harvesting or processing can affect wine quality [152,153]. Household infestations may cause damage to carpets and furniture and trigger allergic reactions [154]. It is evident, however, that no single biological control agent possesses all desirable attributes, and zero risk is not an attainable goal. When evaluating the efficacy and risks of biological control programs, consideration must extend beyond the functional characteristics of the control agent itself to encompass the ecological context of its application [155]. Prior to introducing variegated ladybirds into different regions, local natural prey and predator populations should be surveyed and assessed to determine suitability for release and appropriate release rates.

Research on artificial rearing of ladybirds has been ongoing for nearly seventy years. To date, fully replacing natural prey with artificial diets for large-scale, multi-generational reproduction remains unachievable. Future research could focus on the following directions: Developing a large-scale rearing system combining “alternative prey + key nutrients”, targeting essential nutrients that promote ovary or reproductive system development to overcome the current reproductive limitations in large-scale production while maintaining feasibility and nutritional completeness. Designing artificial diet formulations based on the nutritional composition of natural prey (aphids and their honeydew) and using metabolomics and other technologies to precisely identify bioactive factors that stimulate feeding, development, and reproduction in *H. axyridis*. Utilizing nanocarrier encapsulation, microencapsulation, and other advanced diet processing techniques to enhance the stability and commercial applicability of artificial diets. With further advances in insect nutrition and synthetic biology, artificial diets for *H. axyridis* are expected to overcome current limitations, providing efficient and low-cost natural enemy resources for sustainable pest management.

## Figures and Tables

**Table 1:** Effect of different alternative hosts for rearing *Harmonia axyridis*.

Alternative Prey (AP)				Control Check (CK)	Effects of Ladybirds’ Feeding (AP vs. CK)		Reference
**Order**	Species	Life Stage	Treatment		Larvae	Adult	
Lepidoptera	*Ephestia kuehniella*	Eggs	UV Treatment 25 min + freezing 15 days	*Acyrthosiphon pisum*	Developmental duration: 14.1 d vs. 14.8 d Survival rate: 85% vs. 80%	Adult body weight: 24.4 mg vs. 29.6 mg	[35]
		Eggs	UV-treated and frozen for storage	*Acyrthosiphon pisum*	Developmental duration: 14.1 d vs.14.5 d Survival rate: 96.7% vs. 76.2% *	Fecundity/Total eggs laid: 393 eggs vs. 154 eggs * Adult body weight: 36.1 mg vs. 29.4 mg *	[36]
		Eggs	Laboratory rearing	*Aphis gossypii*	Developmental duration: 14.9 d vs. 14.3 d Survival rate: 91.7% vs.91.7%	Fecundity/Total eggs laid: 379.6 eggs vs. 302.1 eggs	[37]
		Eggs	Frozen	*Cinara atlantica*	Developmental duration: 22.8 d vs. 22.4 d	Egg hatchability: 7.2 d vs. 6.9 d Fecundity/Total eggs laid: 555.5 eggs vs. 747.5 eggs * Adult body weight: 24.7 mg vs. 25.8 mg *	[38]
		Eggs	Frozen	*Brevicoryne brassicae*	Developmental duration: 22.8 d vs. 22.5 d	Egg hatchability: 7.2 d vs. 6.1 d - Fecundity/Total eggs laid: 555.5 eggs vs. 641.0 eggs * Adult body weight: 24.7 mg vs. 25.5 mg *	[38]
		Eggs	Frozen	*Aphis gossypii*	Developmental duration: 14.07 d vs. 15 d * Survival rate: 82.1% vs. 87.7%	Egg hatchability: 21.3 d vs. 13.9 d * Fecundity/Total eggs laid: 387.8 eggs vs. 236.3 eggs * Adult body weight: 33.6 mg vs. 27.8 mg *	[39]
	*Corcyra cephalonica*	Eggs	Frozen eggs	*Myzus persicae*	Survival rate: 0% vs. 87.7% **	Fecundity/Total eggs laid: 0 vs. 236.3 eggs **	[40]
	*Sitotroga cerealella*	Eggs	Fresh	*Sitotroga cerealella* frozen eggs	Developmental duration: 11.2 d vs. 13.4 d Survival rate: 84% vs. 80% *	Egg hatchability: 8.1 d vs. 9.5 d Fecundity/Total eggs laid: 715.3 eggs vs. 606.6 eggs * Adult body weight: 26.8 mg vs. 23.1 mg *	[41]
		Eggs	Stored at 4 °C	*Chaitophorus populeti*	Developmental duration: 11.3 d vs. 9.3 d * Survival rate: 72.1% vs. 90% **	Egg hatchability: 31.1 d vs. 7.4 d * Fecundity/Total eggs laid: 135.6 eggs vs. 732.7 eggs *	[42]
		Eggs		*Aphis gossypii*	Developmental duration: 19.7 d vs. 14.3 d * Survival rate: 47.9% vs. 91.7% *	Egg hatchability: 17.3 d vs. 7.3 d * Fecundity/Total eggs laid: 51.6 eggs vs. 302.1 eggs ** Adult body weight: 19.9 mg vs. 23.0 mg *	[37]
		Eggs	Frozen eggs	*Lipaphis erysimi*	Developmental duration: 39.5 d vs. 25.3 d * Survival rate: 56.7% vs. 77.7% *	Egg hatchability: 18.3 d vs. 11.0 d * Fecundity/Total eggs laid: 391.9 eggs vs. 929.3 eggs	[43]
	*Pectinophora gassypiella*	Eggs	Fresh	*Sitotroga cerealella*	Developmental duration: 19.0 d vs. 18.0 d Survival rate: 94.0% vs. 91.0%	Fecundity/Total eggs laid: lower * Adult body weight: 27.0 mg vs. 23.0 mg *	[44]
	*Bombyx mori*	Larvae	Live	Aphids	Developmental duration: 9.2 d vs. 9.5 d Survival rate: 98.3% vs. 95.0%	Fecundity/Total eggs laid: 85.5 eggs vs. 297.6 eggs * Adult body weight: 29.6 mg vs. 29.3 mg	[45]
	*Mythimna separata*	Eggs	Frozen eggs	*Aphis craccivora*	Developmental duration: 9.9 d vs. 9.7 d	Egg hatchability: 12.3 d vs. 8.9 d * Fecundity/Total eggs laid: 987.1 eggs vs. 1175.5 eggs *	[46]
Hymenoptera	*Trichogrammatidae*	Pupa	Refrigerated	*Myzus persicae*	Developmental duration: 12.3 d vs. 10.3 d ** Survival rate: 81.2% vs. 84.6%	Fecundity/Total eggs laid: 0 vs. 404.1 eggs ** Adult body weight: 20.2 mg vs. 22.4 mg **	[40]
Coleoptera	*Hypera postica*	Larvae	Live	*Acyrthosiphon pisum*		Fecundity/Total eggs laid: 0 vs. 508.5 eggs **	[47]
Anostraca	*Artemia salina*	Cyst	With shell	*Aphis gossypii*	Developmental duration: 19.0 d vs. 15.0 d * Pupal weight: 27.0 mg vs. 40.0 mg * Emergence rate: 72.5% vs. 78.8% -	Fecundity/Total eggs laid: 151.8 eggs vs. Not measured Egg hatchability: 25.1% vs. Not measured	[48]

Note: Symbols indicate non-significant gaps (-), Significant difference (* *p* < 0.05) and highly significant difference (** *p* < 0.01) in biological parameters in the treated group compared to the control group.

**Table 2:** *Harmonia axyridis* artificial diets based on liver.

Artificial Diets (AD)		Control Check (CK)	Effects of Ladybirds’ Feeding (AD vs. CK)		Reference
Basic Components	Other Components		Larvae	Adult	
Pig liver	Sucrose, honey, silkworm pupal powder, water	Aphids	Developmental duration: 21.3 d vs. 21.1 d Pupal weight: 28.9 mg vs. 21.7 mg	Pre-oviposition period: 26.7 d vs. 13 d Lifespan: 52.9 d vs. 55.1 d Fecundity/Total eggs laid: 152.7 eggs vs. 200.9 eggs	[72]
Pig liver	Isio 4 oil, olive oil, sucrose, glycerol, amino acid solution (including tyrosine, histidine, arginine, ethanolamine), yeast extract, Vanderzant vitamin mix	*Ephestia kuehniella* eggs	Egg hatchability: 27.6% vs. 91.5% * Developmental duration: 25.5 d vs.15.3 d *	Pre-oviposition period: 13.5 d vs. 6.0 d * Fecundity/Total eggs laid: 47 eggs vs. 483 eggs * Adult body weight: 21.0 mg vs. 38.5 mg *	[73]
Pig liver	Honey	*Chaitophorus populeti*	Developmental duration: 14.83 d vs. 14.5 d - Pupal duration: 3.6 d vs. 4.0 d *		[74]
Pig liver	Honey, sucrose, water, preservatives (potassium sorbate, penicillin, etc.)	*Chaitophorus populeti*	Developmental duration: 14.3 d vs. 9.3 d * Survival rate: 11.6% vs. 90.0% *	Pre-oviposition period: 29.0 d vd. 7.4 d * Fecundity/Total eggs laid: 68.5 eggs vs. 732.7 eggs * Adult body weight: 25.5 mg vs. 27.0 mg *	[42]
Pig liver	Flaxseed oil, olive oil, sucrose, glycerol, amino acid solution, yeast extract, vitamin mix	*Ephestia kuehniella* eggs	Developmental duration: 20.5 d vs. 12.5 d * Survival rate: 31.7% vs. 86.0% *	Pre-oviposition period: 13.6 d vs. 7.3 d * Lifespan: 93 d vs. 75 d * Fecundity/Total eggs laid: 37 eggs vs. 324 eggs * Adult body weight: 20.1 mg vs. 31.8 mg *	[75]
Pig liver	Fly larval powder, yeast extract, sucrose, honey	*Acyrthosiphon pisum*	Survival rate: 91% vs. 100% - Developmental duration: 17.0 d vs. 13.7 d *	Adult body weight: 31.0 mg vs. 26.0 mg *	[76]
Pig liver	Honey, sucrose, olive oil, insect-specific multivitamins	*Lipaphis erysimi*	Developmental duration: 8.2 d vs. 14.1 d * Survival rate: 45.3% vs.77.7% *	Pre-oviposition period: 17.2 d vs. 11.7 d * Lifespan: 78.6 d vs. 95.5 d * Fecundity/Total eggs laid: 262.3 eggs vs. 929.3 eggs *	[43]
Pig liver	Honey, vitamin C, sugars, royal jelly, glucose, trehalose	Aphids	Developmental duration: 24.5 d vs. 21.5 d *	Pre-oviposition period: 11.8 d vs. 7.9 d - Fecundity/Total eggs laid: 527.7 eggs vs. 830 eggs *	[77]
Pig liver powder	Yeast, sucrose, honey, vegetable oil, potassium sorbate solution, rapeseed pollen, distilled water	*Acyrthosiphon pisum*	Survival rate: 88.3% vs. 96.7% - Development time: 25.6 d vs. 14.5 d **	Adult body weight: 20.4 mg vs. 32.1 mg ** Lipid content: significantly higher than in the aphid-fed group	[78]
Pig liver	Egg, pork, brown sugar, vitamins and preservatives (based on [79] improvement)	*Acyrthosiphon pisum*	Survival rate: 82.2% vs. 71.1% * Developmental duration: 16.2 d vs. 12.5 d *	Pre-oviposition period: 19.8 d vs. 8.9 d * Lifespan: 103.6 d vs. 85.7 d * Fecundity/Total eggs laid: 23.5 eggs vs. 437.3 eggs *	[80]
Pig liver	Egg white, egg, wheat germ, honey, brewer’s yeast, refined sugar, beef, milk, etc. (AD), multivitamins, sodium benzoate, parabens	*Aphis gossypii*	Survival rate: 65.6% vs. 87.7% * Developmental duration: 21.7 d vs. 15.0 d *	Adult body weight: 18.0 mg vs. 27.8 mg * Pre-oviposition period: 40.7 d vs. 13.9 d * Fecundity/Total eggs laid: 60.7 eggs vs. 236.3 eggs *	[39]
Pig liver	Whole egg liquid, pork, yeast extract, royal jelly, sucrose, amino acid mixture, Wess salts, olive oil, soybean oil, vitamin B complex, vitamin C, cephalosporin, methylparaben, potassium sorbate, fava bean leaves, etc.	*Acyrthosiphon pisum*	Survival rate: 66.4% vs. 81.7% Developmental duration: 18.7 d vs. 12.6 d *	Adult body weight: 20.6 mg vs. 23.6 mg * Fecundity/Total eggs laid: 0 vs. 1158.3 eggs *	[81]
Chicken liver	Sucrose, honey, brewer’s yeast, casein hydrolysate, soybean oil, salt mixture [82], Vanderzant vitamin supplement, bulb starch	*Sitotroga cerealella eggs*	Developmental duration: 22.5 d vs. 18.0 d * Survival rate: 94.0% vs. 93.0% -	Adult body weight: 27.5 mg vs. 27.0 mg -	[44]
Beef liver	Shrimp, beef, egg yolk, honey, sucrose, yeast extract, vitamin powder, olive oil, etc.	*Acyrthosiphon pisum*	Developmental duration: 13.9 d vs. 9.0 d *	Adult body weight: 17.2 mg vs. 24.0 mg *	[83]

Note: Symbols indicate non-significant gaps (-), significant difference (* *p* < 0.05), and highly significant difference (** *p* < 0.01) compared to the given ladybird after the given life parameter.

**Table 3:** Sugar composition in aphid honeydew (%).

Components	Research Object										
	*Aulacorthum solani*	*Macrosiphum euphorbiae*	*Sitobion avenae*	*Rhopalosiphum padi*	*Myzus persicae*	*Metopeurum fuscoviride*	*Brachycaudus cardui*	*Trama troglodytes*	*A. fabae*	*Macrosiphoniella tanacetaria*	
	*Solanum tuberosum*		*Triticum aestivum*		*Solanum tuberosum*	*Triticum aestivum*	*Tanacetum vulgare*				
	[147]						[148]				
Fructose	20	15.70	28.3	68.4	41.2	43.2	11.8	11.4	28	8.3	10.6
Glucose	9.20	2	5.9	14.8	7.1	5.1	6.7	8.4	2.5	11.9	25.9
Sucrose	44.50	60	45.3	5.9	30.9	26	3.2	2.2	4.9	2.5	1.9
Trehalose	4.80	2.8	1.8	1.7	6.2	7.1	14.2	20.1	8.2	8.6	44.6
Raffinose	0	0	0	0	0	0	0.1	0	0.3	8.5	0.1
Maltose	1.50	0.9	1.5	2.7	2.1	2.3	1.1	5.6	1.3	6	0.8
Isomaltulose	18.30	18	16.7	5.6	12.1	15.6	62.7	52.4	54.9	54.1	16.6
Melibiose	0	0	0	0.1	0	0	/	/	/	/	/
Mannitol	1.60	0.6	0.4	0.7	0.4	0.6	/	/	/	/	/
Sorbitol	0.10	0	0.1	0.1	0	0.1	/	/	/	/	/

Note: The slash (/) indicates that the component was not determined in the reference(s).

**Table 4:** Amino acid composition in aphid honeydew (%).

Components	Research Object						
	*Aphis nerii*		*Metopeurum fuscoviride*	***Brachycaudus cardui***	***Trama troglodytes***	***Aphis fabae***	***Macrosiphoniella tanacetaria***
	*Asclepias incarnata*	*Asclepias curassavica*	*Tanacetum vulgare*				
	[92]		[148]				
Glu	12.0	29.0	4.5	6.2	8.9	14.3	4.7
Ser	26.0	47.0	13.8	3.7	11.1	4.1	12.5
Gln	/	/	16.1	43.7	29	15.2	6.2
Asn	/	/	17.3	11.9	15.2	9.9	39.6
Asp	5.0	7.0	3.8	2.5	2.8	2.5	7.3
Pro	23.0	19.0	4	3.1	5.2	5.7	5.2
Val ^a^	3.0	2.0	/	/	/	/	/
Val ^a^/Trp ^a^	/	/	3.2	3.6	1.4	10.3	1.1
Ile ^a^	2.0	0.0	0.8	5.3	5.3	1.6	0.3
Leu	11.0	4.0	1.4	1.7	2.6	1.4	0.3
Phe ^a^	9.0	1.0	2.3	0.8	0	1.8	0.5
Arg ^a^	/	/	2.5	2.5	3.1	4.8	4.7
Gly	/	/	3.1	2.1	1.7	1.8	1.6
Thr ^a^	/	/	9.7	5.2	3.9	0.5	8.9
Tyr	/	/	12.4	6.3	1.4	5.5	4.8
Ala	/	/	2.7	0.9	2.5	1.5	0.6
Met ^a^	/	/	0	0.1	0.5	0	2.3
Cys	/	/	0	0.2	3	0.9	0

Note: The slash (/) indicates that the component was not determined in the reference(s). ^a^ An essential amino acid.

## Data Availability

No new data were created or analyzed in this study. Data sharing is not applicable to this article.

## Abbreviations

The following abbreviations are used in this manuscript:

AP	Alternative Prey
AD	Artificial Diets
CK	Control Check
VG	Vitellogenin
VN	Vitellin
JH	Juvenile Hormone
ZR512	Ethyl 3,7,11 trimethyldodeca-2,4-dienoate
